# The Precipitation of Niobium Carbide and Its Influence on the Structure of HT250 for Automobile Wheel Hubs

**DOI:** 10.3390/ma14206109

**Published:** 2021-10-15

**Authors:** Zhu-Hua Zhou, Sheng-Qiang Song, Robert Cromarty, Yi-Liang Chen, Zheng-Liang Xue

**Affiliations:** 1 The State Key Laboratory of Refractories and Metallurgy, Wuhan University of Science and Technology, Wuhan 430081, China; zhouzhuhua@wust.edu.cn (Z.-H.Z.); chenyiliang@wust.edu.cn (Y.-L.C.); 2 Key Laboratory for Ferrous Metallurgy and Resources Utilization of Ministry of Education, Wuhan University of Science and Technology, Wuhan 430081, China; 3 Department of Materials Science and Metallurgical Engineering, University of Pretoria, Pretoria 0002, South Africa; robert.cromarty@up.ac.za

**Keywords:** niobium carbide, grey cast iron, structure, precipitation

## Abstract

Improving the strength of grey cast iron wheel hubs will improve the safety of automobiles and have a great significance for energy saving and environmental protection. This paper systematically compares the calculation results of Python-based precipitation calculation and JmatPro software simulation with experiments. The results show that with a low mass fraction of niobium (0.098%) cuboid Niobium Carbide (NbC) precipitates do not form in the liquid phase; however, an elongated NbC niobium-rich phase may form during the solidification process and in the solid phase. However, cuboid NbC precipitates can be precipitated from the liquid phase when the niobium mass fraction is higher (0.27%, 0.46%). These results indicate that with the increasing niobium content the amount, particle size, and initial precipitation temperature of niobium carbide precipitated in the matrix structure will increase. According to the observation and statistical analysis of the microstructure, it is found that tensile strength will be improved with an increase in niobium content due to the refinement of the graphite and pearlite interlamellar spacing. In this paper, adding less than 0.32% of Nb to grey cast iron is recommended, considering the comprehensive cost and the effect of niobium in the material structure.

## 1. Introduction

The wheel hub is one of the most critical components on a vehicle. At present, grey cast iron is the key material for the wheel hub of domestic and foreign trucks. Improving the strength of grey cast iron wheel hubs not only improves the safety of automobiles but also has a great significance for energy saving and environmental protection. Grey cast iron is composed of a pearlite matrix, graphite flakes and precipitated carbides [1,2,3]. Due to the relatively simple production process, low cost, high recovery rate, high thermal conductivity, excellent machinability and casting performance, grey iron castings maintain a significant position in the market [4,5,6]. Recently, the method of adding a trace amount of niobium elements to improve the structure and mechanical properties of grey cast iron has received extensive attention [6,7,8].

Niobium can refine graphite flakes and decrease the interlamellar spacing of pearlite, thereby effectively improving the tensile strength, fatigue properties and impact toughness of grey cast iron [8,9,10]. The present research found that the addition of niobium will cause NbC to precipitate, strengthen the structure and improve the mechanical properties of grey cast iron since the affinity of niobium with carbon is much greater than that of iron with carbon [11,12,13]. Zhou [14] researched and analyzed the effect of niobium elements on grey cast iron. Their results showed that most of the niobium forms a niobium-rich phase embedded in the metal matrix. The bar-shaped niobium-rich phases are formed at the grain boundaries, and the massive NbC will be precipitated before the liquidus. Therefore, it is of great significance to explore the existing form of niobium and the effect of precipitated NbC on the structure of grey cast iron.

However, most researchers investigated a wide range of niobium contents and did not systematically simulate the precipitation of niobium carbide in the liquid phase and the solidification process. The main purpose of the present work was to investigate the morphological changes in NbC precipitation and its influence on microstructure and tensile strength using the thermodynamic calculation of NbC precipitation. We investigated the theoretical basis and reference for morphological changes in NbC precipitation by comparing the calculation results of Python-based precipitation and JmatPro software simulation with experiments.

## 2. Materials and Methods

### 2.1. Materials

The raw material in the paper is HT250 grey cast iron (niobium-free cast iron) provided by Hubei Yemao Technology Co., Ltd. (Xiangyang, China). A ferroniobium alloy from Beijing Remtech Technology Co., Ltd. (Beijing, China) (Nb 66.97%, Al ≤ 3.0%, Si ≤ 3.0%, Fe ≥ 27.03%, with a particle size range of 1 mm to 5 mm), was used to produce microalloyed cast iron. During the composition design, the alloys with a niobium content of 0%, 0.1%, 0.3% and 0.5% were added. The ferroniobium alloys and HT250 grey cast iron were added as cold charging in the induction furnace; therefore, the dissolution of niobium was largely sufficient. The 75FeSi alloy (Tianjin Zhuxin metal material Co., Ltd., Tianjin, China) with a size range of 0.1 mm to 0.5 mm was used as the inoculant in the inoculation treatment, and was added as 0.5% of the mass of the molten iron.

### 2.2. Methods

A total of 200 g of niobium-free cast iron and ferroniobium was put into the φ30 mm × 50 mm crucible, and the crucible was placed in a small, 35 KW vacuum-induction furnace (Zhengzhou CY Scientific Instrument Co., Ltd., Zhengzhou, China). The niobium-free cast iron and ferroniobium were smelted and held at a temperature of 1773 K for 20 min to ensure the ferroniobium was completely dissolved. The 75FeSi inoculant was added to the melt under stirred conditions. The furnace was then allowed to cool to room temperature at a rate of 20 °C/min. The size of the sample after being smelted was φ30 mm × 30 mm.

The size of the metallographic observation sample was 15 mm × 15 mm × 15 mm, which was wire-cut from a φ30 mm × 30 mm grey cast iron sample after smelting. Standard metallographic techniques were used to grind and polish the samples to a 1.5 µm finish. The graphite morphology was determined by optical microscopy (OM) (Hubei Shangguang Instrument Co., Ltd., Wuhan, China) using a Zeiss Axioscope A1 microscope (Hubei Shangguang Instrument Co., Ltd., Wuhan, China). The specimens of the scanning electron microscopy were chemically etched on the surface by 4 vol.% nitric alcohol solution. A Zeiss EVO MA10 scanning electron microscope (SEM) (Beijing Opton Optical Technology Co., Ltd., Beijing, China) equipped with energy dispersive X-ray spectroscopy (EDS) (Beijing Opton Optical Technology Co., Ltd., Beijing, China) was used to measure pearlite interlamellar spacing and analyze the phases present. ImageJ software (National Institutes of Health, ImageJ 1.53c, Bethesda, MD, USA) was used to process the data and statistics of the shape and size of graphite and the interlamellar spacing of pearlite.

Sample carbon and sulfur were analyzed using a CS996 infrared carbon–sulfur analyzer (Hefei Demai Testing Instrument Co., Ltd., Hefei, China). All other elements were analyzed using an IRIS Advantage ER/S ICP-AES instrument (Thermo Jarrell Ash Corporation, Boston, MA, USA). The composition of the grey cast iron samples is shown in Table 1.

## 3. Simulation Calculation of Niobium Carbide Precipitation

### 3.1. NbC Precipitation Calculation Based on Python

The precipitation reaction for the formation of NbC in molten iron is given in Equation (1) [15]:

(1) $$\left[\mathrm{Nb}\right]+\left[C\right]{=\mathrm{NbC}}_{\left(S\right)}\;\Delta G^{\theta }=-182,490+96.99T\;$$

**Figure 1:**
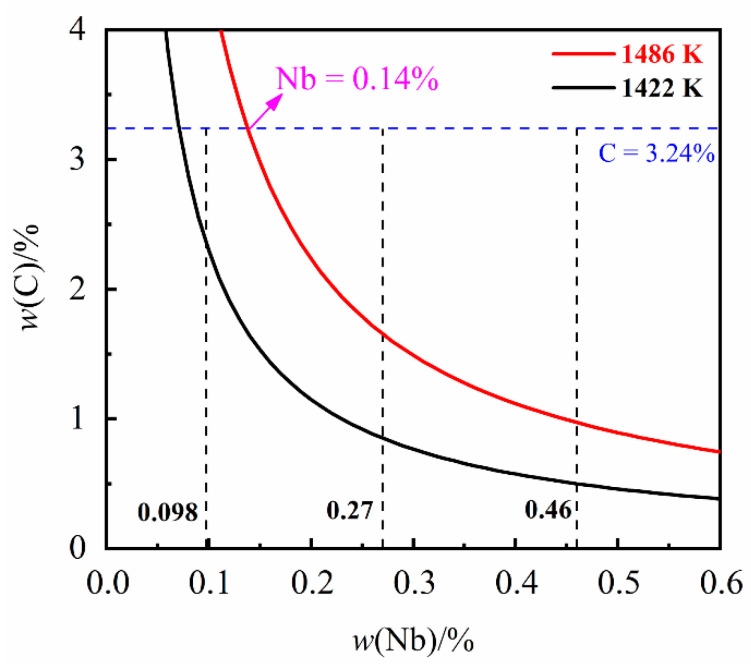
The solubility product curves of NbC in liquidus (1486 K) and solidus (1422 K).

In the light of the solidification process, the equation for calculating the equilibrium concentration product (*K*_NbC_) is shown in Equation (2):

(2) $$\log K_{\mathrm{NbC}}=5.0664-9532.6437/T$$

where *K*_NbC_ is the equilibrium activity product corresponding to the precipitation of NbC; *T* is the temperature (K). The NbC solubility product curves at the temperature of liquidus (1486 K) and solidus (1422 K) were obtained according to Equation (2) and Table 2, and are displayed in Figure 1.

Figure 1 shows the solubility limits for dissolved niobium and carbon at the liquidus (1486 K) and solidus (1422 K) temperature of the grey cast iron. From Figure 1 it can be seen that, for a carbon content of 3.24%, the NbC will be precipitated in the liquid while Nb content has exceeded 0.14%. It can be found that NbC can be precipitated in the liquid while the niobium content is 0.27% and 0.46%. On the contrary, while the niobium content is 0.098% NbC will be precipitated at temperatures between the solidus and liquidus.

When the temperature continues to drop towards the solidification process, the value of *K*_NbC_ is decided by the solidification front temperature *T_s–l_*, which is expressed by Equation (3) [16]:

(3) $$T_{s-l}=T_{0}-\frac{T_{0}-T_{l}}{1-g\frac{T_{l}-T_{s}}{T_{0}-T_{s}}}$$

where *T_l_* is the cast iron liquidus temperature (1486 K); *T_s_* is the cast iron solidus temperature (1422 K); *T*_0_ is the melting point of pure iron (1811 K) and *g* is solidification fraction. The actual concentration product of Nb and C on the front of molten iron solidification can be expressed by Equation (4):

(4) $$Q_{NbC}=f_{\mathrm{Nb}}\times f_{C}\times w(\mathrm{Nb})\;\times w\left(C\right)$$

where *f*_Nb_ and *f*_C_ are the activity coefficients of Nb and C, respectively, which can be calculated by the interaction coefficient in Table 2; *w*(Nb) and *w*(C) are the actual concentrations of Nb and C on the solidification front (mass%). The values of the first-order interaction coefficient *e_i_^j^* of elements involved in the computation are listed in Table 2 [16,17], where *i* are the elements of C and Nb; *j* are the solute elements.

The Ohnaka model assumes that the Nb element and the C element diffuse finitely in the solid phase and completely diffuse in the liquid phase. The model equation is represented by Equation (5) [16]:

(5) $$w(i)=w{\left(i\right)}_{0}\times {\left[1-(1-\beta k_{i})g\right]}^{\frac{k_{i}-1}{1-\beta k_{i}}}$$

where *w*(*i*) is the actual content of the solute element *i* at the solidification front (mass%) and *w*(*i*)_0_ is the initial content of the solute element *i* (mass%), while *k_i_* is the equilibrium partition coefficient for the solute element *i* between γ-Fe phase and the molten steel. In this paper, the value of *k_i_* was calculated by the “Equilib” module integrated into the software Fact Sage 8.0 based on the chemical composition in Table 1. The “Festeel” database was also applied. The equilibrium distribution coefficient of Nb and C used in the calculation are listed in Table 3 [18,19]; *β* is the back-diffusion parameter, which is calculated by Equation (6) in the Ohnaka model for columnar dendrites as follows [18]:

(6) $$\beta =\frac{4\alpha }{1+4\alpha }$$

where *α* is the Fourier parameter for a solute element and can be obtained by Equation (7) [18]:

(7) $$\alpha =\frac{4D_{i}{}^{\gamma }\left(T_{l}-T_{s}\right)}{R_{c}\times \lambda^{2}}$$

where *D_i_*^γ^ is the diffusion coefficient of the solute element (cm^2^/s), which as used in this paper will be listed in Table 3 [18,19]; *R_C_* is the cooling rate (°C/s); λ is the secondary dendrite arm spacing (cm), which varies with cooling conditions and carbon content. The empirical relationship formula of the secondary arm spacing λ is obtained by Equation (8) [17]:

(8) $$\lambda =143.9\times R_{C}{}^{-0.3616}\times w{\left(C\right)}_{0}{}^{\left(0.5501-1.996\times w{\left(C\right)}_{0}\right)}$$

**Figure 2:**
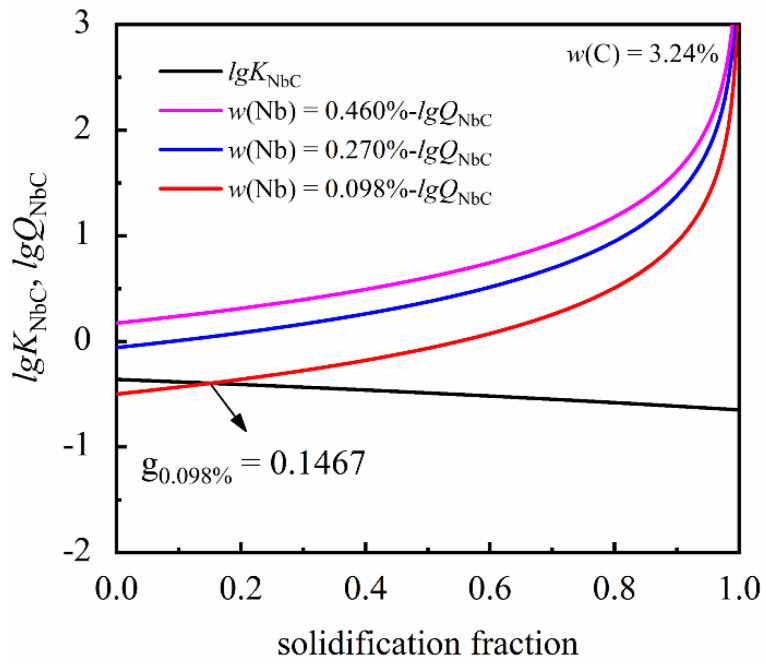
The variations of actual concentration product and equilibrium solubility product during solidification.

After formulating the carbide precipitation model, a Python program, using the data in Table 2 and Table 3, was used to calculate the conditions required to precipitate NbC at the solidification front. The variations of concentration product during solidification are shown in Figure 2. It is clearly shown that when the solidification fraction reaches 0.1467 within the condition of 3.24% C and 0.098% Nb, the actual concentration product reaches the equilibrium solubility product, which results in NbC precipitation during solidification. On the other hand, whilst a higher content of niobium (0.27% Nb, 0.46% Nb) is added to grey cast iron, NbC will be precipitated in the liquid phase.

As the temperature cools to the γ-Fe temperature region, the precipitation solubility product of NbC in γ-Fe can be expressed by Equation (9) [20]:

(9) $$\mathrm{lg}\left(\mathrm{Nb}\right){\left(C\right)}_{\gamma }=2.96-\frac{7510}{T}$$

The solubility product of NbC in γ-Fe can be calculated to be 5.31 × 10^−5^ to 4.77 × 10^−3^ in the γ-Fe temperature range (1038 K to 1422 K). When the temperature drops to the γ-Fe to α-Fe transition temperature the precipitation solubility product of NbC in α-Fe is represented by Equation (10) [20]:

(10) $$\mathrm{lg}{\left(\mathrm{Nb})(C\right)}_{\alpha }=5.43-\frac{10960}{T}$$

**Figure 3:**
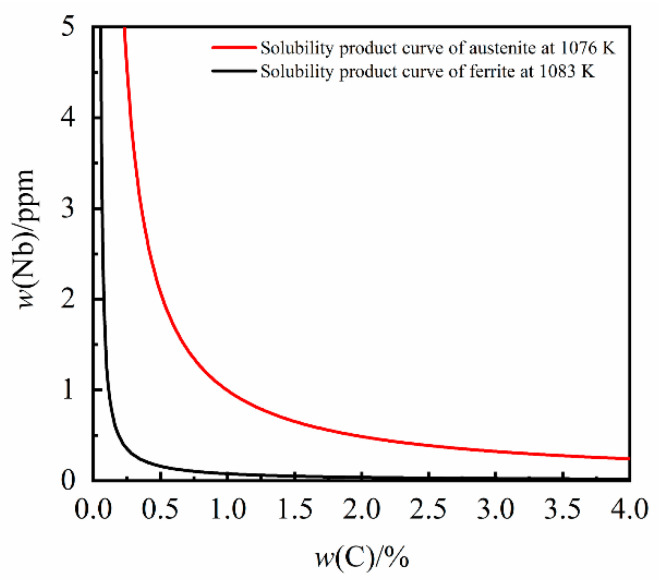
The solubility product curves of NbC in the solid phase.

The solubility product of NbC in α-Fe can be calculated to be less than 1.75 × 10^−5^ in the α-Fe temperature range (below 1076 K). The solubility product curves of the second phase of NbC in different solid phases are shown in Figure 3. Whilst the iron matrix structure of grey cast iron changes from γ-Fe phase to α-Fe phase, the solubility product of NbC will drop sharply.

The solubility product of NbC decreases with a decrease in temperature in both γ-Fe and α-Fe. It is evident that the precipitation of NbC will be enhanced in the low temperature α-Fe phase. However, due to the influence of kinetic factors, the amount of NbC precipitated is limited.

As noted above, it was found that although NbC cannot be precipitated in the liquid within the condition of 3.24% C and 0.098% Nb, the NbC niobium-rich phase can be precipitated during the solidification process and the phase transition process. On the other hand, NbC can be precipitated in the liquid phase when the niobium content is 0.27% and 0.46%.

### 3.2. JMatPro Simulation Results and Analysis

**Figure 4:**
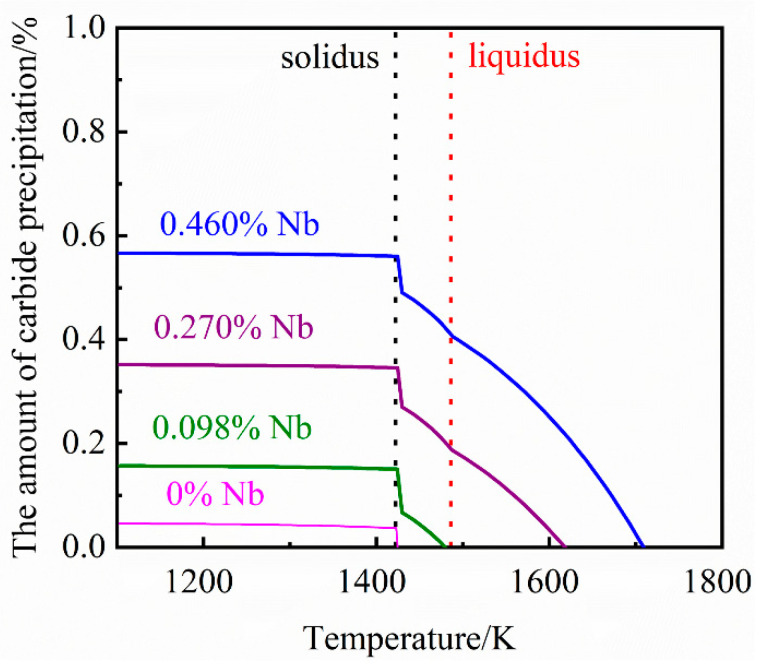
The relationship between different niobium contents and the amount of carbide precipitation.

**Figure 5:**
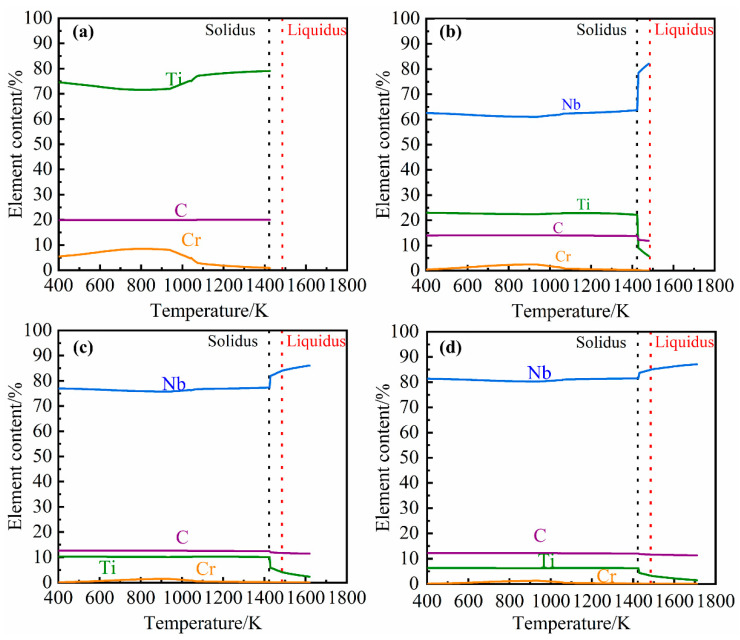
Relationship between MC-type carbide precipitate composition and temperature and alloy niobium content. (**a**) 0% Nb; (**b**) 0.098% Nb; (**c**) 0.27% Nb; (**d**) 0.46% Nb.

Previous studies have proven [21,22] that the NbC second phase in grey cast iron is an MC-type carbide. Under the composition of this study, the simulation and analysis of MC-type carbides precipitated from grey cast iron containing 0%, 0.098%, 0.27% and 0.46% Nb was carried out in simulation using JMatPro software. The influence of different niobium content on the amount and composition of carbide precipitation are demonstrated in Figure 4 and Figure 5, respectively. From Figure 4, it can be seen that the amount of carbide formed and the temperature of initial precipitate formation increases with increasing niobium content.

As seen in Figure 5, when there is no niobium in the cast iron the MC-type carbide precipitates consist mainly of TiC. As the niobium concentration in the cast iron is increased, the niobium content of the precipitates will increase. Carbide precipitates only form in the liquid phase when there is sufficient niobium (0.27% and 0.46%) in the cast iron. However, it can be found that the tendency of carbides to precipitate will increase rapidly and the niobium content in carbides will drop sharply in the temperature range of solidus and liquidus. The reason for this phenomenon is that cuboid carbides precipitated from the liquid phase contain a higher amount of niobium than the elongated precipitates formed and precipitated during solidification and the solid phase.

## 4. Results and Discussion

### 4.1. Existing Form and Distribution of Niobium

#### 4.1.1. Solid Solution in the Grey Cast Iron Matrix

Niobium can be solutionized in α-Fe and pearlite structures. The amount of solid solution will increase with the increase in the niobium content. However, only the trace niobium will be dissolved into the matrix structure of grey cast iron [23]. Although the solid solubility of niobium at room temperature is extremely limited, the trace amount of solid solution still plays a strengthening role in solid solution and maintains this solid solution strengthening effect at higher temperatures.

#### 4.1.2. Elongated NbC Niobium-Rich Phase

**Figure 6:**
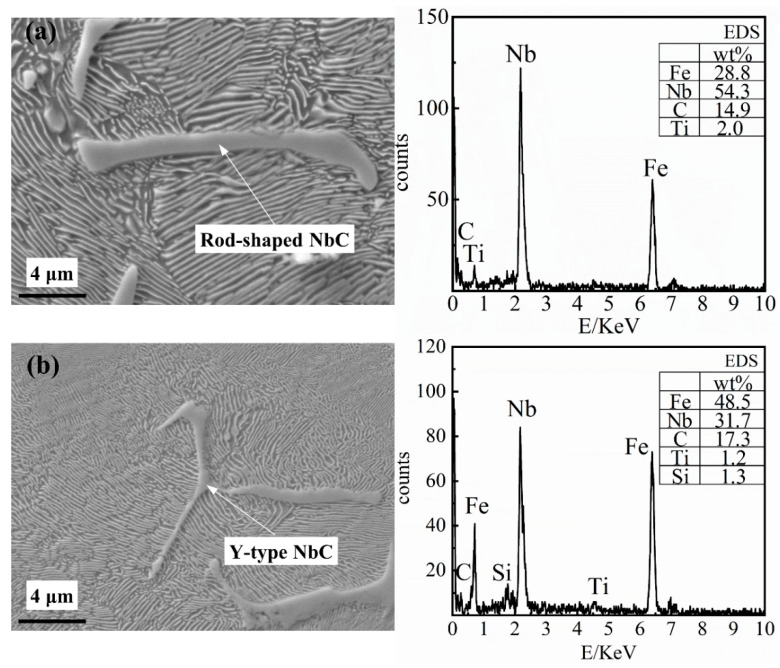
The energy-dispersive X-ray spectroscope (EDS) results of the elongated NbC niobium-rich phase, (**a**) Rod-shaped NbC; (**b**) Y-type NbC.

The shapes of the elongated NbC niobium-rich phase generally include rod-shaped (as shown in Figure 6a), Y-type (as shown in Figure 6b) and V-type. This elongated NbC niobium-rich phase type of carbide, which is commonly found in niobium-containing cast iron and niobium-containing alloy steel, is relatively slender, and the length of each branch arm is generally 2–20 μm. When adding a small amount of niobium to the grey cast iron, the elongated NbC niobium-rich phase can be precipitated during the solidification process and the phase change process after solidification, which can produce a second phase strengthening effect on the metal matrix structure.

It can be seen from Figure 6 that Fe, Nb and C account for more than 95% of the mass with Nb accounting for 30% to 60% of the mass. The main precipitation in the matrix structure is the elongated NbC niobium-rich phase whilst the niobium content is low (0.098% Nb). Therefore, the elongated NbC niobium-rich phase is precipitated during the solidification process or the phase change process after solidification. With the increasing niobium content, the number of the elongated NbC niobium-rich phases precipitated in the matrix will increase. In the meantime, increasing the niobium content will result in an increase in branch arm length and thickness of the NbC precipitates. The microstructure observation is consistent with the simulation results.

#### 4.1.3. Cuboid NbC Precipitate

**Figure 7:**
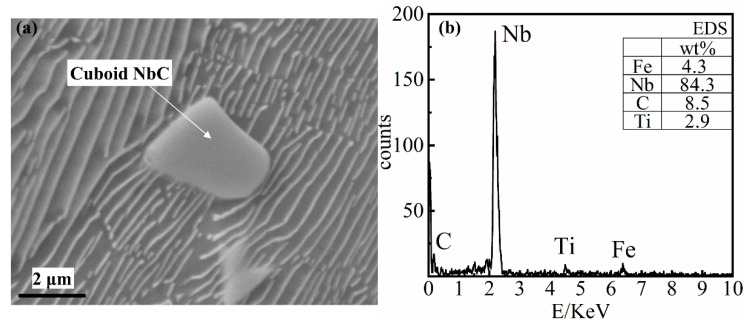
The energy-dispersive X-ray spectroscope (EDS) result of Cuboid NbC precipitate. (**a**) the SEM image of the cuboid NbC; (**b**) the EDS of the cuboid NbC.

The size of cuboid NbC precipitates (as shown in Figure 7) is generally 1–10 μm. This type of second phase, which is mainly embedded in the pearlite structure and has a relatively regular shape, can also produce a second phase strengthening effect on the metal matrix structure.

As shown in Figure 7, Nb and C account for more than 92.5% of the mass, with Nb accounting for more than 80% of the mass. The cuboid NbC precipitates cannot be precipitated whilst the niobium content is low (0.098% Nb). When the niobium content is high (0.27% and 0.46%) both cuboid NbC precipitates and elongated NbC precipitates are found in the matrix. By comparing calculation and simulation, it can be determined that the cuboid NbC precipitates are precipitated from the liquid phase. As the concentration of niobium increases, the amount of the cuboid NbC precipitates will increase, and the particle size will increase accordingly.

### 4.2. The Influence on the Graphite Structure

In grey cast iron, graphite has both detrimental and beneficial effects: on the one hand, the sharp corners of graphite inside the grey cast iron will cause stress concentration, which reduces the strength and results in a decrease in these properties. On the other hand, due to its great lubricity, excellent thermal conductivity and outstanding shock absorption performance, graphite brings correspondingly good performance to grey cast iron.

**Figure 8:**
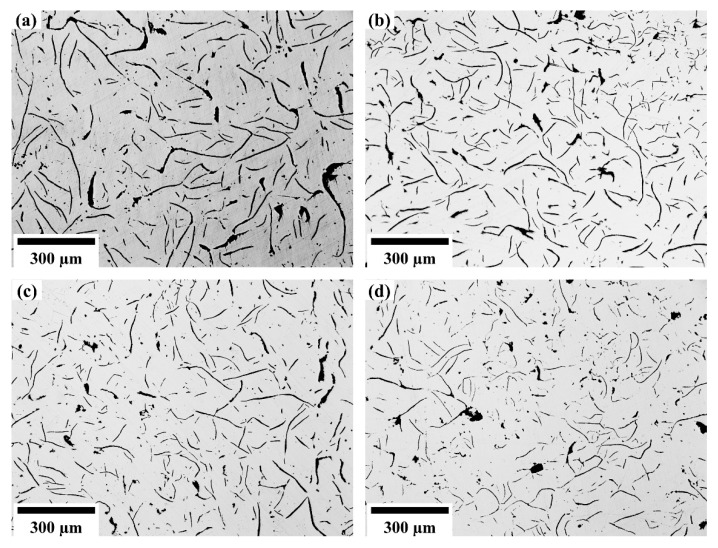
The graphite structure with different niobium content. (**a**) 0% Nb; (**b**) 0.098% Nb; (**c**) 0.27% Nb; (**d**) 0.46% Nb.

**Figure 9:**
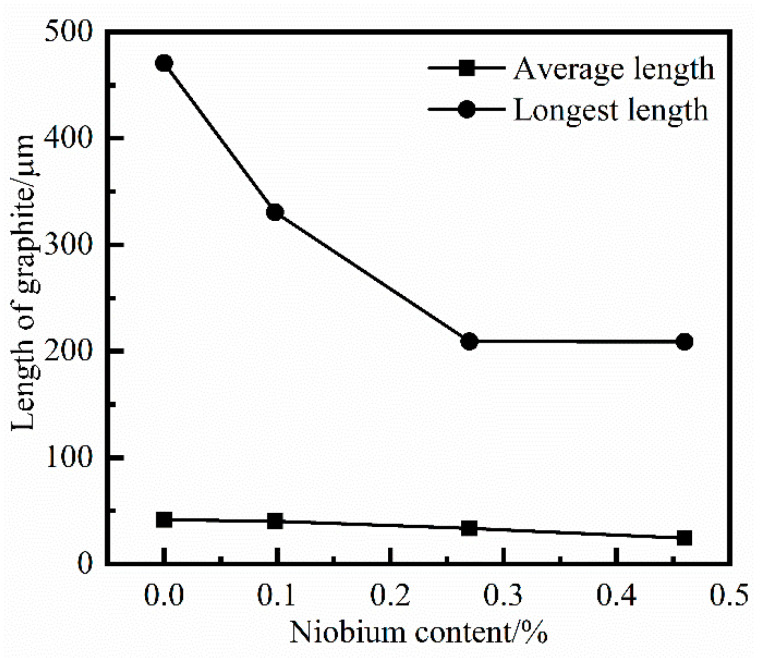
The statistics on the length of graphite with different niobium content.

After the specimens were ground and polished, the shape, size and related distribution of the graphite structure of the grey cast iron were observed with a metallurgical microscope. Figure 8 shows the optical micrograph of the graphite structure with different niobium contents. The influence of niobium on the graphite morphology was assessed using optical microscopy. The graphite in twenty metallographic pictures of each sample was measured and statistically analyzed according to the National Standards of the P. R. China (GB/T 7216-2009) [24]. The average length and the longest graphite flake in each of the twenty micrographs were calculated. Results are presented in Figure 9.

From the comparison of Figure 8 and Figure 9, the addition of niobium cannot change the type of graphite structure, which is still Type A graphite in the grey cast iron. However, with increasing niobium concentration, both the longest length and average length of graphite were reduced. The longest length of graphite was transformed from 470.84 μm to 208.90 μm when the niobium content was increased from 0% to 0.46%. Furthermore, the average length of graphite was also reduced from 41.46 μm to 24.41 μm.

According to the trend of the influence of adding niobium to the graphite structure, it is clear that the effect of refining the length of graphite was most obvious when the amount of niobium in grey cast iron is 0.098%. As the content of niobium increased, the effect of niobium on the refinement of the length of graphite was constantly attenuated, and the trend of graphite refinement also slowed down.

The refinement of graphite will have a beneficial impact on the tensile strength of grey cast iron. However, when the content and quantity of the graphite are too large, the sharp corners of the graphite flakes will cause stress concentration and result in a decrease in the tensile strength. The results of [25] indicate that 0.08% Nb decreases the extent of graphitization in grey cast iron. In this case, the tensile strength was transformed from 671 MPa to 746 MPa as the niobium content increased from 0% to 0.08%. Therefore, the refinement effect of niobium on graphite needs to be controlled within an appropriate range.

### 4.3. The Influence of Niobium on Pearlite Matrix Structure

**Figure 10:**
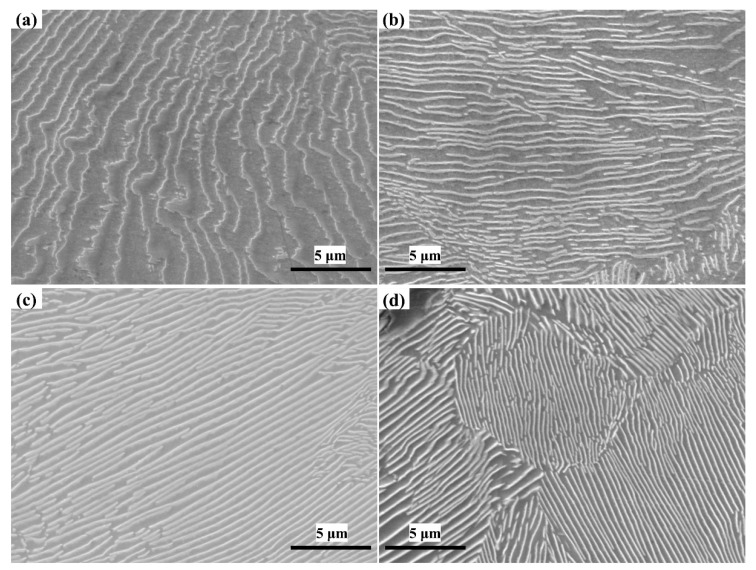
The pearlite structure with different niobium content. (**a**) 0% Nb; (**b**) 0.098% Nb; (**c**) 0.27% Nb; (**d**) 0.46% Nb.

Compared with other grey cast irons, pearlite matrix grey cast iron, which has higher strength, greater hardness, and less brittleness, is a more competitive material in the casting market. Typical SEM micrographs of the four sample types are presented in Figure 10.

The field of view in the scanning electron microscope picture was randomly selected for the sample. The pearlite lamellar spacing can be calculated by Equation (11) [26]:

(11) $$S_{0}=\frac{\pi D}{nM}$$

where *S*_0_ is the interlamellar spacing of pearlite; *D* is the diameter of the selected circle; *M* is the magnification of the scanning electron microscope; *n* is the number of points where the selected circle and the cementite sheet intersect. The tests of each sample will be performed in 20 random fields of view and the average value taken to calculate the pearlite interlamellar spacing.

**Figure 11:**
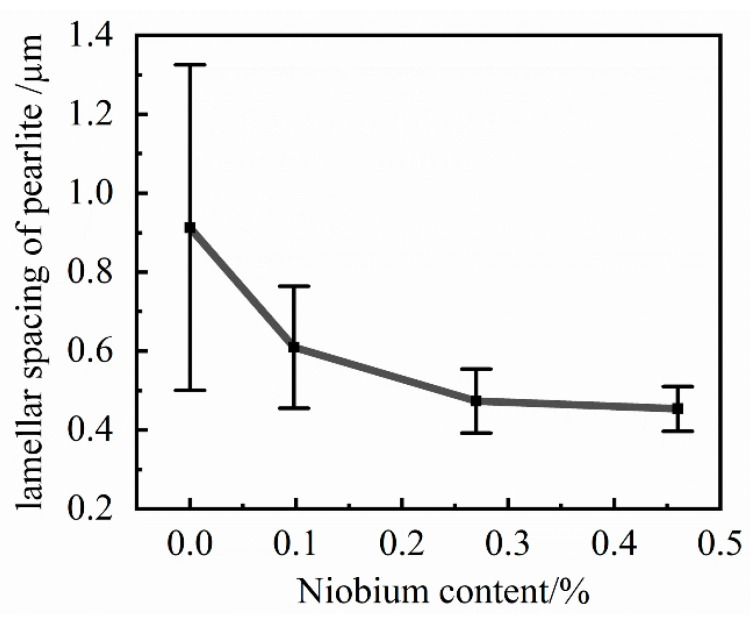
The refinement trend graph of the lamellar spacing of pearlite.

The refinement trend graph of pearlite lamellar spacing is shown in Figure 11. The addition of niobium to the cast iron resulted in a decrease in the interlamellar spacing of pearlite. However, the influence on the interlamellar spacing of pearlite also constantly weakened. The refinement effect of niobium on pearlite lamellar spacing needs to be controlled within an appropriate range. The statistical results show that with the increasing niobium content, the formation of the internal structure of grey cast iron also tends towards a pearlite matrix structure with smaller interlamellar spacing. Consequently, a pearlite structure with a relatively large interlamellar spacing cannot exist in grey cast iron with higher niobium content. The niobium element had a relatively obvious tendency to refine the pearlite structure with a larger interlamellar spacing but had a smaller effect on the pearlite structure with a smaller interlamellar spacing.

In order to improve its mechanical properties, two ways to change the pearlite structure have been proffered. One way is to reduce the grain size of pearlite, and the other is to reduce the interlaminar spacing of the pearlite matrix. The reasons for the reduction of the pearlite lamellar spacing are following: on the one hand, niobium reduces the pearlite transformation temperature and increases the degree of subcooling, which ultimately leads to a decrease in the lamellar spacing of pearlite. On the other hand, the drag effect of the niobium element dissolved in the metal matrix structure on the carbon atoms makes the carbon atoms unable to migrate over a large range, and finally leads to the reduction of the pearlite lamellar spacing.

The performance of pearlite depends to a large extent on the size of the interlamellar spacing of pearlite. With the decrease in the pearlite interlamellar spacing, the strength of pearlite will be increased, which ultimately leads to the improvement of the tensile strength of grey cast iron. Chen et al. [25] found that when 0.08% Nb was added to grey cast iron, the pearlite interlamellar spacing structure was reduced from 1.04 μm to 0.87 μm, which ultimately led to an increase in strength of 11.2%. The refinement effect of niobium on pearlite lamellar spacing thus needs to be controlled within an appropriate range.

### 4.4. The Influence on the Tensile Strength

The changes in the graphite flake size and the pearlite interlamellar spacing influence the tensile strength of the cast iron, and these properties will be discussed in the following section.

**Figure 12:**
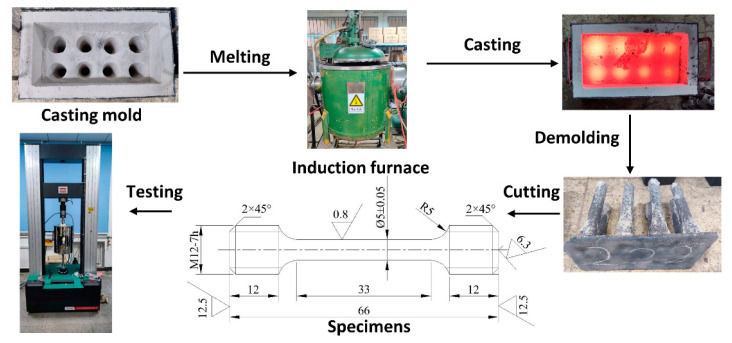
Tensile test specimen preparation and testing.

To test the influence of niobium on the tensile strength of HT250 grey cast iron, samples containing 0% and 0.32% niobium were prepared. A smelting process diagram is shown in Figure 12. The alloys were melted in a vacuum induction furnace and cast into bars. The bars were then machined to produce round tensile test specimens with a diameter of 5 mm and a gauge length of 33 mm. Samples were then tested using a UTM5305G microcomputer-controlled testing machine, with a tension rate of 0.1 mm/min. Since grey cast iron is a brittle material, the strain rate used in the tensile test was low [27]. A total of eight samples of each alloy composition were tested.

**Figure 13:**
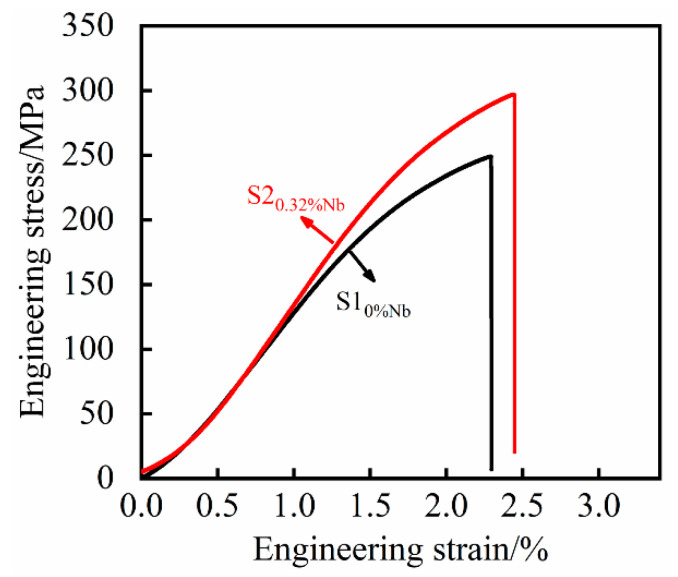
The engineering stress–strain curves.

Figure 13 shows the stress–strain curves of the tensile process. Tensile testing experiments (Figure 13) revealed that the fracture mechanism of the samples was brittle. It is clear that as the content of niobium increases, the slope of stress–strain curves and the strength also increase. It can be indicated that the addition of Nb also increases the elastic modulus of the grey cast iron. Ultimately, a greater stress was required to cause a brittle fracture of the material, thereby showing an increase in the tensile strength of the specimen.

In addition to the tensile tests, metallographic samples of 0% Nb and 0.32% Nb composition were prepared. As for the previous alloy compositions, OM was used to assess the size of the graphite flakes and SEM microscopy was used to measure the interlamellar spacing of the pearlite. In the observation of the structure of cast iron containing 0.32% Nb, the graphite structure and the pearlite structure are presented in Table 4. As can be seen from Table 4, the graphite flake size and pearlite interlamellar spacing for the 0.32% niobium alloy were consistent with the results from previously prepared samples. Comparing the room temperature tensile strength of the two different contents of niobium, as shown in Table 4, it can be shown that the room temperature tensile strength of grey cast iron with 0.32% Nb was increased by 19.24% compared with grey cast iron with no addition of niobium.

At present, the price of ferroniobium is 180 CNY/kg. The addition of 0.32% Nb increases the cost of grey cast iron by 0.86 CNY/kg; however, the strength increases by 19.24%. It is obvious that comprehensive economic benefits are thus obtained. In view of the effect on the refinement results and cost factors, it is recommended that the addition amount of niobium should be less than 0.32%.

## 5. Conclusions

In this paper the precipitation of niobium carbide and its influence on the structure and strength of HT250 grey cast iron were investigated. Conditions necessary for precipitation of NbC were investigated using JmatPro- and Python-based simulations. The properties of the graphite flakes and pearlite were measured for a range of niobium contents, while the tensile strength was measured for alloys containing 0% and 0.32% Nb. It can be concluded that:

(1) For grey cast iron containing 3.24% C, NbC can only precipitate in the liquid at higher (0.27% Nb and 0.46% Nb) niobium contents. At the higher niobium concentrations, cuboid NbC will precipitate in the liquid. On the contrary, at lower (0.098% Nb) niobium concentrations elongated NbC will precipitate during the initial stage of solidification and the phase-transition process.
(2) Niobium in the cast iron may be in solid solution, in the form of cuboid NbC precipitates or in the form of cuboid NbC precipitates. When the niobium content is low (0.098% Nb), the elongated NbC niobium-rich phase is the main form; cuboid precipitates only form at higher niobium concentrations (0.27% and 0.46% in this work). The number and size of the precipitates increase with increasing niobium concentrations.
(3) Niobium in the cast iron does not change the shape of the graphite flakes nor the amount of pearlite formed. However, the addition of niobium to the cast iron will reduce the size of the graphite flakes and reduce the interlamellar spacing of the pearlite. Changes in the graphite and pearlite result in an increase in the tensile strength of the cast iron.
(4) Considering the cost of niobium and the limited influence of high concentrations of niobium on the pearlite structure it is recommended that a maximum of 0.32% niobium be added to the cast iron.

## Figures and Tables

**Table 1:** The chemical composition of the samples after smelting (% by mass).

Number	C	Si	Mn	P	S	Cr	Ti	CE *	Nb
1	3.25	2.08	0.72	0.016	0.15	0.33	0.036	3.95	<0.01
2	3.24	2.10	0.72	0.017	0.17	0.33	0.036	3.95	0.098
3	3.24	2.09	0.71	0.017	0.15	0.34	0.036	3.94	0.270
4	3.24	2.09	0.70	0.016	0.15	0.34	0.036	3.94	0.460

* Carbon equivalent (CE) = *w*(C) + 1/3[*w*(Si) + *w*(P)].

**Table 2:** Values of first-order interaction coefficient of elements in molten iron [16,17].

*e_i_^j^*	C	Si	Mn	P	S	Cr	Ti	Nb
C	0.14	0.08	−0.012	0.051	0.046	−0.024	0	−0.06
Nb	−0.49	-	0.0028	-	−0.047	−0.011	0	0

**Table 3:** Values of equilibrium partition coefficients as well as diffusion coefficients in austenite [18,19].

Elements	*k_i_*	*D_i_*^γ^/(cm^2^·s^−1^)
C	0.46	0.076 e^(−32160/(R*Ts–l*))^
Nb	0.04	0.83 e^(−63690/(R*Ts–l*))^

**Table 4:** The composition as well as strength of tensile test samples (% by mass).

Number	C	Si	Mn	P	S	Nb	Longest Graphite (μm)	Average Graphite (μm)	Pearlite Interlamellar Spacing (μm)	Strength (Mpa)
S1	3.18	2.00	0.68	0.016	0.10	0	468.27	42.57	0.9034	251.0
S2	3.17	2.00	0.68	0.017	0.10	0.32	210.58	34.39	0.4871	299.3

## Data Availability

Not applicable.
